# Diagnostic Coding Intensity among a Pneumonia Inpatient Cohort Using a Risk-Adjustment Model and Claims Data: A U.S. Population-Based Study

**DOI:** 10.3390/diagnostics12061495

**Published:** 2022-06-19

**Authors:** Ruchi Mishra, Himadri Verma, Venkata Bhargavi Aynala, Paul R. Arredondo, John Martin, Michael Korvink, Laura H. Gunn

**Affiliations:** 1 School of Data Science, University of North Carolina at Charlotte, Charlotte, NC 28223, USA; rmishra5@uncc.edu (R.M.); hverma1@uncc.edu (H.V.); vaynala@uncc.edu (V.B.A.); parredon@uncc.edu (P.R.A.); 2 ITS Data Science, Premier, Inc., Charlotte, NC 28277, USA; john_martin@premierinc.com (J.M.); michael_korvink@premierinc.com (M.K.); 3 Department of Public Health Sciences, University of North Carolina at Charlotte, Charlotte, NC 28223, USA; 4 Department of Primary Care and Public Health, School of Public Health, Imperial College London, London W6 8RP, UK

**Keywords:** ICD-10-CM diagnosis, coding intensity, pneumonia, Poisson additive model, risk adjustment

## Abstract

Hospital payments depend on the Medicare Severity Diagnosis-Related Group’s estimated cost and the set of diagnoses identified during inpatient stays. However, over-coding and under-coding diagnoses can occur for different reasons, leading to financial and clinical consequences. We provide a novel approach to measure diagnostic coding intensity, built on commonly available administrative claims data, and demonstrated through a 2019 pneumonia acute inpatient cohort (N = 182,666). A Poisson additive model (PAM) is proposed to model risk-adjusted additional coded diagnoses. Excess coding intensity per patient visit was estimated as the difference between the observed and PAM-based expected counts of secondary diagnoses upon risk adjustment by patient-level characteristics. Incidence rate ratios were extracted for patient-level characteristics and further adjustments were explored by facility-level characteristics to account for facility and geographical differences. Facility-level factors contribute substantially to explain the remaining variability in excess diagnostic coding, even upon adjusting for patient-level risk factors. This approach can provide hospitals and stakeholders with a tool to identify outlying facilities that may experience substantial differences in processes and procedures compared to peers or general industry standards. The approach does not rely on the availability of clinical information or disease-specific markers, is generalizable to other patient cohorts, and can be expanded to use other sources of information, when available.

## 1. Introduction

The International Classification of Diseases—Clinical Modification, Tenth Revision (ICD-10-CM), is a standard for disease classification widely used for coding medical diagnoses. Diagnostic coding describes patients’ conditions and is utilized throughout inpatient stays. It is essential to accurately capture these codes as they are critical to adequate healthcare service delivery and outcomes, and the associated reimbursement process [1]. Hospitals receive reimbursements for inpatient services based on the Medicare Severity Diagnosis-Related Group’s (MS-DRG’s) estimation [2]. A base MS-DRG is assigned to each hospitalization based on the patient’s primary diagnosis, and diagnosis codes are identified for discharge and payment purposes [3]. These include secondary diagnoses recorded during an inpatient stay, with more diagnoses recorded often associated with higher hospital reimbursements.

There is a financial incentive to record diagnoses codes for inpatient visits, as it can lead to higher hospital reimbursement payments. This includes those with excess coding, also known as over-coding [4]. However, over-coding, whether deliberate or unintentional, is considered fraud and can result in an audit and subsequent penalties [4]. Inappropriate coding of diagnoses or healthcare services can lead to risks concerning patient safety if such miscoding translates into inappropriate clinical follow-up upon discharge, with associated costs oftentimes underestimated [5,6]. Tsopra et al. found diagnostic coding inaccuracy rates as high as 58% [7]. In some cases, clinicians’ misdiagnoses, or inappropriate coding by personnel, result in unreliable coding, leading to inaccurate patient medical records that may never be corrected. Thus, the detection of inappropriate coding practices can support enhanced patient health, during and after the inpatient visit, and enhance organizational outcomes by helping to identify where there could be true variations in care that need to be addressed [8]. There is an increasing need to study diagnostic coding intensity to provide healthcare organizations with a better understanding of their clinical coding and potential areas of improvement in coding practices [9]. While the literature tends to focus on health outcomes, there is limited research on the actual coding intensity of secondary diagnoses, which can be a relevant factor to identify and enhance coding practices within facilities, with the potential to improve associated health outcomes.

The Medicare Advantage (MA) payment system uses risk scores to assign a risk category based on diagnostic coding [9]. Coding intensity, in this context, is the difference between the scores that beneficiaries would obtain in the MA program and their fee-for-service program scores, and it is used to adjust the provider’s payment [9].

From a regulatory and quality perspective, it has been found that when pneumonia diagnosis codes are recoded to sepsis or respiratory failure, which could be a legitimate change, performance measures in the U.S. Centers for Medicare and Medicaid Services (CMS) programs such as the Hospital Readmissions Reduction Program (HRRP) can be improved, highlighting the need for accurately documenting and coding patient conditions [10]. Rothberg et al. had similar findings, reporting an association between pneumonia mortality rates and the definitions that hospitals use to identify pneumonia admissions, highlighting how coding practices and definitions can influence overall hospital performance [11]. Lindenauer et al. found that the hospitalization rate increased with additional pneumonia secondary diagnoses (with principal diagnoses of sepsis or respiratory failure), stressing the relevance of proper accounting for secondary diagnoses [12]. Other studies support the need to address changes in pneumonia coding practices as they relate to reduced pneumonia mortality rates [11,13].

There is a strong need for generalizable diagnostic coding intensity models that can provide an industry-based expected count of diagnoses upon discharge and identify potential cases of insufficient or excessive diagnostic coding intensity. In order for these approaches to be of use to quality control personnel and for assessing hospital quality performance, they must allow for inter-facility comparisons and not be reliant on complex or sparse data, which constrain the use across different disease-specific patient cohorts. This study provides an easy-to-implement diagnostic coding intensity model built on commonly available administrative claims data that include both patient- and facility-level characteristics for risk adjustment, though flexible to include other types of patient information, such as EHRs. Our approach is demonstrated through a motivating example of a pneumonia inpatient cohort and is easily extrapolatable to other disease conditions.

## 2. Materials and Methods

### 2.1. Data

De-identified data from Premier, Inc.’s private all-payor administrative claims database was used in this study [14]. Observations for 184,398 acute inpatient hospital stays of first patient visits with discharge dates in 2019 for those diagnosed with pneumonia were extracted. The cohort of interest was identified using pneumonia-associated MS-DRG codes: 193 (simple pneumonia and pleurisy with major complication or comorbidity (MCC)); 194 (simple pneumonia and pleurisy with complication or comorbidity (CC)); and 195 (simple pneumonia and pleurisy without CC/MCC). The data extract contains the following: (1) response variable representing the total count of secondary diagnoses throughout the inpatient stay; (2) patient-level characteristics, which include patient age, sex, race, primary payor, point of origin, patient discharge status, ICD-10-CM principal diagnosis code, MS-DRG code, length of stay, and Agency for Healthcare Research and Quality (AHRQ) overall tract summary social vulnerability index; (3) facility-level characteristics including Case Mix Index (CMI) (rounded for de-identification purposes), teaching status, academic status, urban/rural status, ownership status, size (i.e., bed count), and U.S. Census Bureau regional division; and (4) admission month.

### 2.2. Statistical Analysis

Descriptive statistics were calculated for all variables. A complete case analysis was performed to exclude missing values, which comprised 0.94% of the observations. Variables containing categories with overly low observed frequencies (<0.1%) were grouped into combined categories defined as “other” across multiple variables. For non-ordered categorical variables, categories with the highest frequencies were defined as the reference categories for modeling. For the ordered categorical variables, a facility size (bed count in ranges) of 1–100 beds was defined as the reference category, and for age category, 85 years and older was defined as the reference group.

A Poisson additive model (PAM) was selected to model additional diagnoses counts, though alternatives are possible when over-dispersion may be present. The linear predictor in the model includes a spline that allows for a smoother, potentially non-linear association between the length of stay covariate and the outcome. While most of our covariates were categorical in nature for non-identifiability (e.g., bed counts), the spline approach can be extended to other covariates if more granular data are available. For example, age was accessible in ranges, but if available in continuous form, it would be sensible to use a spline for it, since pneumonia disproportionally affects those who are younger as well as older, and processes for diagnosis of patients could be non-linearly dependent on age. Equation (1) represents the PAM for the counts of additional ICD-10-CM diagnosis codes as a function of patient-level characteristics

D[i]~Poi(λ[i]), where log(λ[i]) = α1 + β*PL[i] + s(log(LOS[i])), (1)

where D[i] represents the observed additional diagnosis counts for patient i, Poi() represents the Poisson distribution, and λ[i] represents the latent, individual-specific mean rate of additional diagnoses. The log-mean rate for individual i, log(λ[i]), is assumed to be linearly associated with: an overall mean level α1; a set of multiplicative coefficients **β** with corresponding patient-level covariates (PL[i]), except length of stay (LOS[i]); and, a thin plate spline (s), which is applied to the sole continuous covariate log-LOS for patient i (log(LOS[i])).

Excess coding intensity (ECI) for each patient i was estimated using Equation (2) as the difference between the observed counts of additional diagnoses for each patient and the PAM-derived expected value ($\hat{\lambda }$) of the counts conditional on the patient-level covariates using Equation (1). This metric is assumed to be normally distributed (though alternative choices are possible), denoted as N(), with mean linearly related to facility-level characteristics FL[i] through a vector of coefficients **θ**, as well as a common error term σ^2^ denoting the variability unexplained by these facility-level characteristics

(2) $$\mathrm{ECI}[i]\;\overset{\mathrm{def}}{=}\;(D[i]-\;\hat{\lambda }[i])\sim N(\theta *\mathrm{FL}[i],\;\sigma^{2}).$$

An adjusted excess coding intensity (AECI) metric was defined for each patient visit i as the regression residual comprised by the difference between the (patient-level-adjusted) ECI metric and fitted values further adjusted by facility-level covariates

(3) $$\mathrm{AECI}[i]\;\overset{\mathrm{def}}{=}\;\mathrm{ECI}[i]-\hat{\theta }*\mathrm{FL}[i].$$

This second metric, AECI[i], extracts the variability in the estimated ECI of patient i that cannot be explained by the additional set of facility-level characteristics FL[i] associated with the facility that patient i attended.

This dual metric approach allows for sequentially calculating patient-level-adjusted (using metric ECI) and patient plus facility-level-adjusted (using metric AECI) inter-facility comparisons of ECI. In instances where facility-level differences in coding intensity are not admissible, the former may be used for comparisons, whereas the latter may be used when different facility-level characteristics can be reasonably expected yet they should be discounted. Both measures aim to extract idiosyncratic variability in coding intensity of diagnoses unexplained by known sources of variability.

Facility rankings by AECI were calculated and extreme values were identified. Quarterly U.S. maps were generated to visualize AECI and explore seasonality by region to also identify temporal variations in diagnosis coding practices. R version 4.1.0 was used for statistical analysis.

## 3. Results

### 3.1. Descriptive Statistics

Table 1 contains descriptive statistics for all variables from 182,666 complete case, unique, inpatient, pneumonia-related hospital admissions, with the outcome (count of secondary diagnoses throughout the inpatient stay) distribution depicted in Figure S1. The average length of stay was 4.09 (standard deviation, SD 3.53) days, which was log-transformed to address heavy skewness, as demonstrated in Figure S2. The mean AHRQ social vulnerability index was 0.54 (SD 0.24). Approximately half of the patients (n = 91,299) were aged 70 and older. The majority of patients self-identified as white (140,060; 76.70%), and over half of the patients were female (98,828; 54.1%). More individuals were insured by traditional Medicare (77,108; 42.2%) than any other primary payor source within this dataset. Additionally, Figure S3 portrays the primary payor by race and displays how some variables may experience multicollinearity to various degrees. Most patients’ point of origin were from non-healthcare facilities (153,451; 84.0%), and nearly two-thirds of patients were discharged to either home or self-care (115,553; 63.3%). The most common ICD-10-CM principal diagnosis was J18, which is pneumonia caused by an unspecified organism (129,404; 70.8%), and the most common MS-DRG was 193, which is simple pneumonia and pleurisy with major complication or comorbidity (92,239; 50.5%).

Most of the patients attended facilities that did not have a teaching (148,656 patients; 81.4%) or an academic (161,362 patients; 88.3%) designation and were identified as urban facilities (152,702 patients; 83.6%). The majority of patients attended a facility with a rounded CMI of 2 (100,970 patients; 55.28%). Nearly two-thirds of patients attended hospitals that were private with a voluntary, not-for-profit ownership status (116,090 patients; 63.6%). Patients attended facilities with the most common bed count range of 201–300 beds (37,147 patients; 20.3%). The majority of patients attended facilities located in the South Atlantic Census regional division (49,091 patients; 26.9%), and the highest number of admissions was observed in March (22,458; 12.3%).

### 3.2. Model Outcomes

Adjusted incidence rate ratios (IRRs) and corresponding 95% confidence intervals (CIs), as well as p-values associated with each patient characteristic, are reported in Table 2. The adjusted IRR for the AHRQ overall tract summary is 0.95 (95% CI 0.94, 0.95), indicating that each additional unit of the AHRQ social vulnerability index is associated with a 5% lower incidence rate of coding additional diagnoses, upon accounting for all other patient variables. Younger patients were generally associated with lower IRRs. For example, patients under one year of age had an adjusted IRR of 0.36 (95% CI 0.35, 0.36), indicating a 64% lower incidence rate of coding additional diagnoses than those over 84 years of age (reference group). Those who identify as Asian were associated with a 15% lower incidence rate of coding additional diagnoses than those identifying as White (IRR 0.85; 95% CI 0.84, 0.86). Patients with charity as their primary payor had an adjusted IRR of 0.71 (95% CI 0.70, 0.73), representing a 29% lower incidence rate of coding additional diagnoses when compared to those with traditional Medicare (reference category), upon accounting for all other patient characteristics. Patients referred from other departments within the same facility were associated with a 13% lower incidence rate of coding additional diagnoses than those referred from a non-healthcare facility (reference) with an adjusted IRR of 0.87 (95% CI 0.85, 0.88). Patients with an ICD-10-CM principal diagnosis code of J09 (influenza due to certain identified influenza viruses) had an adjusted IRR of 0.87 (95% CI 0.85, 0.89), indicating a 13% lower incidence of coding additional diagnoses compared to those with J18 (pneumonia caused by unspecified organisms) as their principal diagnosis. Patients with an MS-DRG code of 195 (simple pneumonia and pleurisy without CC) had an adjusted IRR of 0.60 (95% CI 0.60, 0.61), indicating a 40% lower incidence rate of coding additional diagnoses than those with simple pneumonia and pleurisy with major complication or comorbidity (MS-DRG 193, reference).

While assessing goodness of model fit, 39.2% of the variation in additional diagnosis counts can be explained by patient-level characteristics. Finally, the spline for the log length of stay was also significant (*p* < 0.0001) and is displayed in Figure S4. In addition, the root mean square error (RMSE) indicated a 22.05% reduction, or improvement, versus a non-informative mean level, which strongly supports the use of patient characteristics to risk adjust additional diagnoses counts. Figure S5 portrays the excess coding intensity distribution across patient visits.

**Figure 1:**
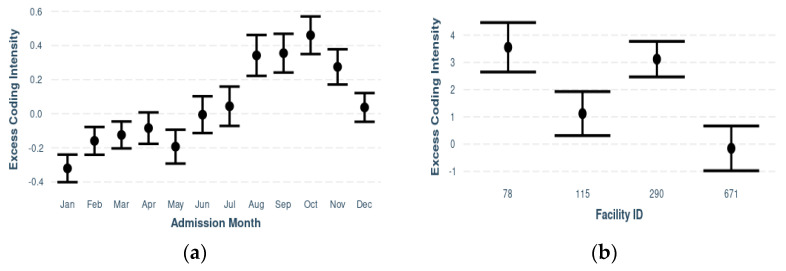
Effect plot illustrating excess coding intensity by admission month (**a**), and effect plot of excess coding intensity estimates for a stratum of hospitals with the same values (modal categories for each variable) for all facility-level characteristics (**b**).

Figure 1a depicts the association between admission month and ECI. Lower values of ECI were observed during the first six months when compared to the latter half of the year. Admission month was statistically significant (*p* < 0.0001). Figures S6–S12 include effect plots of the associations between ECI and each facility characteristic: urban/rural status; ownership status; teaching status; academic status; size (bed capacity); U.S. Census Bureau regional division; and CMI, respectively. When comparing ECI between a stratum of hospitals with equal facility-level characteristics (defined as the most observed categories for each facility-level characteristic), substantial differences in ECI are observed, even upon accounting for patient-level characteristics (Figure 1b).

Table 3 presents the regression coefficients of adjusting the ECI by facility-level characteristics. There is a statistically significant estimated average difference of 0.56 (standard error, SE 0.06) excess coded diagnoses between facilities with versus those without a teaching designation (*p* < 0.0001). Conversely, patients admitted to facilities with an academic designation experience lower ECI levels compared to those attending facilities without an academic designation (*p* < 0.0001), with an average difference of −0.47 (SE 0.07) excess coded diagnoses. There was a significant (*p* < 0.0001) estimated average difference of −1.44 (SE 0.20) excess coded diagnoses between state owned government hospitals and private not-for-profit hospitals. Substantial regional differences were also found, with differences as large as −1.59 (SE 0.05) additional diagnoses per patient visit between geographically adjacent regions (Middle Atlantic versus South Atlantic).

**Figure 2:**
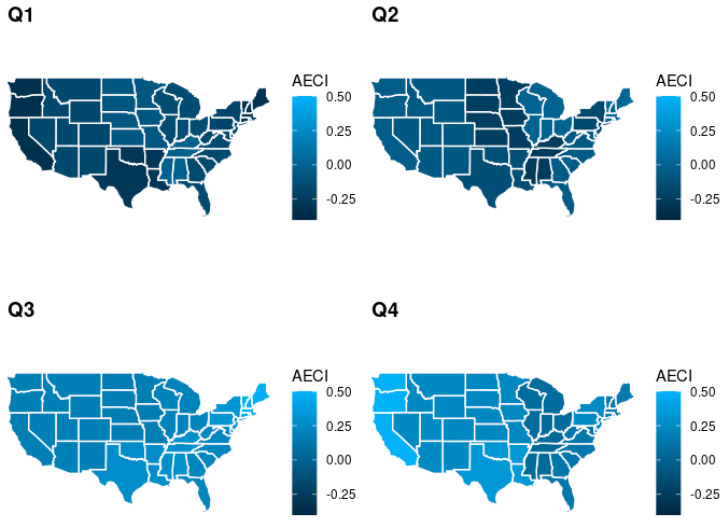
Average adjusted excess coding intensity (AECI) by U.S. Census Bureau regional division within each quarter in 2019.

Figure 2 shows the average AECI by U.S. Census regional division within each quarter during 2019. Lower rates of adjusted excess coding are observed in the earlier part of the year across all regions when compared to the latter parts of the year, demonstrating that monthly seasonal patterns are not region specific. Figure S13 depicts a more detailed heat map of AECI averaged across admission months and U.S. Census Bureau regional divisions.

**Figure 3:**
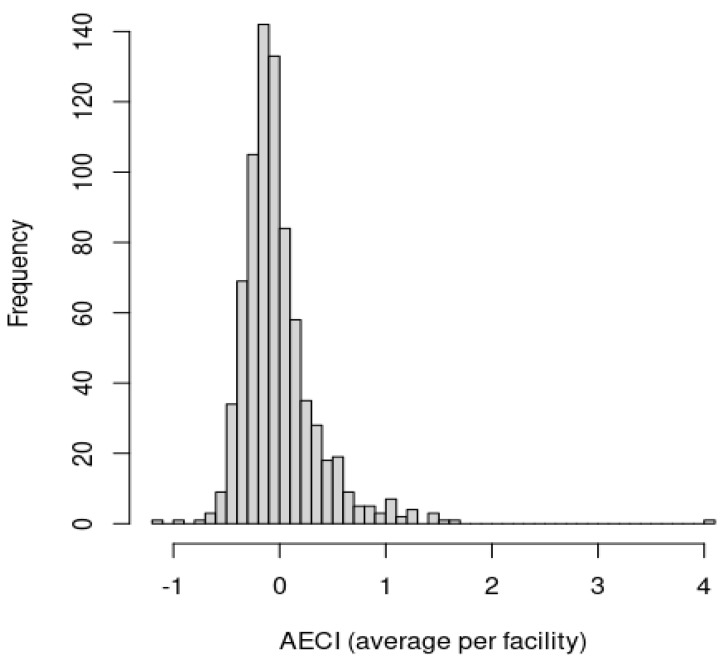
Histogram of the unexplained variability in excess coding intensity (represented by the AECI metric) averaged by facility.

Figure 3 shows a histogram for the unexplained ECI (i.e., AECI), which is the amount of ECI that is not explained by patient- and facility-level characteristics, averaged across patients by facility. Some facilities exhibit large differences versus industry standards in average AECI across patients. For example, a facility with a value of 5 represents an average of five diagnoses per patient more than industry standards for a similar set of patients and facility characteristics. The histogram highlights that most facilities are averaging in the range from −1 to 1 excess coded diagnoses. Average differences of one diagnosis per patient in either direction can quickly compound over patient visits, and demonstrate wide idiosyncratic variability even after averaging across patient visits, which reduces the expected variability due to patient heterogeneity. The ranking of facilities by adjusted excess coding intensity was performed by averaging across patient visits and sorting across facilities, which is visually demonstrated in Figure S14, representing the mean AECI levels by facility (the associated confidence intervals are overly dense to visualize them for a large number of facilities).

## 4. Discussion

We propose a method that can be used to rank facilities according to the unexplained diagnosis coding intensity upon risk adjusting for patient- and facility-level characteristics. Most of the variables considered, both at the facility and patient levels, were significant to explain variability in excess coding intensity. We also observed variation in ECI intra-year. While risk adjustment was necessary and helped explain a large portion of the outcome variability, substantial differences in diagnosis coding intensity were still found beyond values attributable to patient- and facility-level characteristics. AECI was found to vary substantially over time across different geographical regions.

There is limited literature on modeling additional diagnoses using claims data. Melfi et al. showed that a count of unique diagnosis codes was predictive of the length of stay in hospital [15]. von Korff et al. developed a chronic disease score to predict chronic illness using claims data [16]. However, claims data, while readily available across patient cohorts, has not been used, to our knowledge, for risk adjustment and the construction of metrics for modeling excess diagnostic coding.

Younger patients were associated with, in some cases substantially, lower levels of coding than older patients, which may be an indication of differences in prognosis and severity associated with pneumonia, or additional comorbid conditions that tend to increase as people age. It also can be an indication of differences in the clinical meaning of codes by age group [17]. Male patients were found to experience statistically significantly larger incidence rates of diagnosis coding than females but with small magnitudes. Race was found to be largely relevant, with patients of all races but American Indian experiencing lower incidence rates of additional diagnoses than White patients. This may indicate either racial differences in severity or disparities by race, and further research is warranted to attempt to identify causality. Additional units of social vulnerability of patients were associated with lower additional diagnoses counts, which reflects potential disparities by this factor, though, again, there are other confounders that could be present and further research is also warranted. Differences by primary payor are substantial, with charity-based or self-pay patients exhibiting substantially lower additional diagnoses than patients with traditional Medicare. While the patient mix between groups may in part explain these differences, there is a case for further study of whether the differences in additional diagnosis counts are associated with differences in hospital processes and procedures during inpatient stays or related to the MS-DRG payment used by CMS. Large differences in excess diagnoses were identified between patient admissions from a non-healthcare facility versus a different unit within the same hospital as a separate claim. These differences may be expected, with lower diagnosis rates identified for the latter, as these patients may have diagnoses already identified and treated within the separate claim before they arrive to the new unit within the facility; however, it would have been expected that those diagnoses would have been considered comorbid conditions and captured via ICD-10-CM codes upon admission. Differences in excess diagnoses by discharge status can be a reflection of the severity of the patient (a proxy for clinical information). For example, those discharged to hospice–home or hospice–medical facility are likely to be more severe cases than those discharged to home or self-care, as reflected by the 22% and 26% higher incidence rates of diagnoses, respectively. Similarly, the ICD-10-CM code for the principal diagnosis is a relevant clinical factor to explain variability in excess coding intensity, with ICD-10-CM codes representing lower severity, such as influenza-related diagnoses (i.e., J09, J10, and J11), experiencing fewer complications/additional diagnoses than those who enter with pneumonia from an unspecified organism (J18). Interestingly, diagnoses with more specified organisms did not have a higher risk of more intense coding; however, that could have potentially been due to the unknown nature of the infection causing more tests to be performed and leading to more conditions being captured. Not surprisingly, MS-DRGs also account for severity, with patients experiencing major complications/comorbidities also experiencing large differences in incidence rates of additional diagnoses when compared to patients with no complications or non-major complications. Finally, length of stay, which can also be a proxy for severity, was also statistically significant, with a positive spline slope indicating that higher lengths of stay (i.e., more severe cases) were associated with larger counts of additional diagnoses.

Facilities with a teaching status were associated with higher observed ECI. Conversely, facilities with an academic status were associated with lower ECI than those with a non-academic status. There is substantial collinearity between these two variables, with most academic hospitals also being teaching facilities; however, academic facilities are unique in that they tend to have closer ties with academic institutions and often have much larger teaching programs. Hospital ownership status may not differentiate with regards to quality of care, but there are noticeable differences in billing and coding practices [18]. We found very large associations between hospital ownership status and excess coding practices, with local hospitals and state owned facilities substantially under-coding when compared to the reference category of voluntary non-profit private hospitals. Since hospital CMI is accounted for in the analysis, the magnitude of this finding deserves further research, as it may be an indication of structural differences, service offering differences, or simply differences in coding practices by ownership status. More generally, hospitals having district hospital (or authority), local, government (state owned), proprietary, voluntary not-for-profit church-owned, or other voluntary not-for-profit ownership status were associated with statistically lower ECI when compared to those with a voluntary non-profit private ownership status (*p* ≤ 0.0130).

Geographical differences in excess coding intensity were substantial, with patients in the adjacent regions of Middle Atlantic and South Atlantic experiencing very different excess coding intensity levels. It is not possible for us to assert causality and state that Middle Atlantic experiences under-coding or South Atlantic experiences over-coding, but the differences warrant further research into the root causes since averages of 1.59 fewer diagnoses per patient within Middle Atlantic states, upon adjusting for patient characteristics and other facility-level differences, seem to be an excessively large difference. Finally, CMI was also found to be significant, with a CMI of 3, a proxy for a more severe patient mix, associated with higher excess coding intensity, thus explaining this result (note: a CMI of 4, the most severe patient mix, was non-significant, as there were very few facilities with this value).

We observed strong seasonality components in ECI, with substantially lower averages in the first six months of the year in comparison to the last six months. There was a stark increase in ECI from August until October, followed by a sudden decline in the latter part of the year. Since winter months see a higher hospitalization rate [19], and another study has shown a similar pattern in admission months for patients with pneumonia across hospitals [20], then a possible reason for under-coding could be the increased workload on hospital staff, resulting in exhaustion, under-documentation, and under-coding.

### Strengths and Limitations

The reliance on claims data is both a strength and limitation of our approach. We demonstrate that administrative claims data is helpful to explain large portions of the variability in excess coding intensity, and to identify and rank facilities upon adjustment for patient- and facility-level characteristics, allowing for easy implementation across cohorts. However, clinical factors, such as those found in EHRs, could provide additional relevant information to explain diagnostic coding variability. If available, clinical variables could be easily incorporated into our approach as additional covariates. However, these type of data are less readily available, and the inconsistency in data captured is substantially larger. Our model, therefore, serves as a pragmatic solution for quality control and performance assessment when clinical information may not be available, and could be leveraged to achieve higher accuracy if that information becomes available.

The spline adjustment for continuous covariates allows for more flexible assessment of potential associations. Variables such as age, which we could not access in a more granular form from the data provider, would benefit further from this adjustment if available at a more granular level. Some cohorts, such as pneumonia, can experience non-linear prevalence across ages and experience potentially different processes established for the diagnoses of additional conditions during inpatient stays.

Risk adjustment oftentimes suffers from multicollinearity, as many factors are associated with one another. While this presents a challenge during the interpretation of coefficients from the different risk adjustment steps, they will not affect the more relevant outcomes, which are the underlying metrics (overall model fit and subsequent residuals extracted from the model are not affected by multicollinearity). ECI and AECI can be used for facility ranking in the presence of multicollinearity.

While we demonstrate the creation of two different metrics from sequential adjustments by patient- and facility-level characteristics, a single metric combining both sets of factors within the PAM is also possible. The general approach demonstrated in the manuscript relates to the use of regression residuals for the identification of unexplained, risk-adjusted variability. However, multiple possible paths are viable regarding the construction of these metrics.

There could have been a possibility of overlap between symptoms of COVID-19 and symptoms of pneumonia in patients admitted to facilities in the last quarter of 2019. However, it is unlikely that these numbers would have substantially affected our results, as there is no evidence of a large spread of the disease in the population in the U.S. prior to early 2020.

While our proposed approach has been defined against industry averages, if a subset of facilities is known to provide a gold standard process or best practice for accurately recording diagnoses, they could be used as references, and all analyses performed against that set of facilities.

Finally, it is important to note that the proposed metrics do not represent over- or under-performance of hospitals or physicians. They do not represent actual over- or under-coding during inpatient stays either. These metrics represent excess coding above or below what would be expected from a limited set of information available about the patient visits and facilities, all measured against industry standards for these covariates. They are a tool to flag potential divergences against industry standards when combined across multiple patient visits from a facility, but not to demonstrate these differences. In order to draw conclusions about over- or under-coding, facilities would need further analyses of the actual processes for the flagged cohorts. The advantage of our method is that it can be easily applied across facilities and cohorts, providing a “big picture” assessment across diseases, and to flag areas where departures from industry standards may warrant further analysis. Quality control personnel can, in such cases, focus their efforts on assessing these areas, rather than exploring a much larger set of conditions.

## 5. Conclusions

Over- and under-diagnostic coding in hospitals can lead to uncertain measures of clinical performance and have financial, legal, and ethical implications. Demonstrated through a pneumonia patient cohort, two metrics are introduced to risk adjust diagnosis coding intensity for both patient- and facility-level characteristics using solely administrative claims data. The approach can identify hospitals that may be operating outside industry standards, either by flagging facilities with excessive coding practices or those that may be under-coding or under-diagnosing. While clinical information, such as that found in EHRs, may incorporate additional value, quality control personnel can use this approach across cohorts with more readily available data sources as a pragmatic, exploratory tool.

## Figures and Tables

**Table 1:** Summary statistics of patient-level and facility-level characteristics.

Characteristics	Count/Mean (%/SD)
***Outcome***	
Additional Diagnoses (mean, SD)	14.18 (7.74)
***Patient-Level Characteristics***	
Age (years)	
<1	2685 (1.47%)
1–4	6576 (3.60%)
5–9	3213 (1.76%)
10–14	1406 (0.77%)
15–19	1177 (0.64%)
20–24	1459 (0.80%)
25–34	5159 (2.82%)
35–44	7798 (4.27%)
45–54	14,812 (8.11%)
55–59	12,980 (7.11%)
60–64	16,335 (8.94%)
65–69	17,767 (9.73%)
70–74	20,453 (11.20%)
75–79	20,695 (11.33%)
80–84	19,104 (10.46%)
≥85	31,047 (17.00%)
Sex	
Female	98,828 (54.10%)
Male	83,768 (45.86%)
Unknown	70 (0.04%)
Race	
American Indian	1418 (0.78%)
Asian	3319 (1.82%)
Black	22,954 (12.57%)
Pacific Islander	1194 (0.65%)
White	140,060 (76.68%)
Other	10,301 (5.64%)
Unknown	3420 (1.87%)
AHRQ ^1^ Overall Tract Summary (mean, SD)	0.54 (0.24)
Primary Payor	
Charity	659 (0.36%)
Commercial Indemnity	7808 (4.27%)
Direct Employer Contract	359 (0.20%)
Managed Care Capitated	399 (0.22%)
Managed Care Non-Capitated	19,645 (10.75%)
Medicaid–Managed Care Capitated	2620 (1.43%)
Medicaid–Managed Care Non-Capitated	14,695 (8.04%)
Medicaid Traditional	8776 (4.80%)
Medicare–Managed Care Capitated	6160 (3.37%)
Medicare–Managed Care Non-Capitated	35,100 (19.22%)
Medicare Traditional	77,108 (42.21%)
Other Government Payors	2515 (1.38%)
Self-Pay	5319 (2.91%)
Other	1503 (0.82%)
Point of Origin	
Clinic	14,013 (7.67%)
Non-Healthcare Facility Point of Origin	153,451 (84.01%)
Transferred from a Hospital (Different Facility)	7695 (4.21%)
Transferred from Department Unit in Same Hospital	989 (0.54%)
Transferred from Health Facilities	1642 (0.90%)
Transferred from Skilled Facility or Intermediate Care Facility	3888 (2.13%)
Other	175 (0.10%)
Information Not Available	813 (0.45%)
Discharge Status	
Discharged to Home Health Organization	27,954 (15.30%)
Discharged to Home or Self-Care	115,553 (63.26%)
Discharged to Hospice–Home	2416 (1.32%)
Discharged to Hospice–Medical Facility	2021 (1.11%)
Discharged/Transferred to ICF ^2^	1810 (0.99%)
Discharged/Transferred to Other Facility	2095 (1.15%)
Discharged/Transferred to Psychiatric Hospital	325 (0.18%)
Discharged/Transferred to SNF ^3^	21,544 (11.79%)
Discharged/Transferred to Swing Bed	509 (0.28%)
Discharged/Transferred to Other Health Institute Not in List	242 (0.13%)
Discharged/Transferred to a Long-Term Care Hospital	711 (0.39%)
Discharged/Transferred to Another Rehab Facility	2079 (1.14%)
Expired	1963 (1.07%)
Left Against Medical Advice	2547 (1.39%)
Other	897 (0.49%)
ICD-10-CM Principal Diagnosis Code	
J09: Influenza due to certain identified influenza viruses	1004 (0.55%)
J10: Influenza due to other identified influenza virus	25,419 (13.92%)
J11: Influenza due to unidentified influenza virus	1368 (0.75%)
J12: Viral pneumonia, not elsewhere classified	8590 (4.70%)
J13: Pneumonia due to Streptococcus pneumoniae	1977 (1.08%)
J14: Pneumonia due to Hemophilus influenzae	583 (0.32%)
J15: Bacterial pneumonia, not elsewhere classified	13,517 (7.40%)
J16: Pneumonia due to other infectious organisms, not elsewhere classified	523 (0.29%)
J18: Pneumonia, unspecified organism	129,404 (70.84%)
R09: Other symptoms and signs involving the circulatory and respiratory system-as a primary diagnosis code	243 (0.13%)
Other	38 (0.02%)
MS-DRG ^4^ Code	
193: Simple Pneumonia or Pleurisy with MCC ^5^	92,239 (50.50%)
194: Simple Pneumonia and Pleurisy with CC ^6^	66,386 (36.34%)
195: Simple Pneumonia and Pleurisy without CC/MCC	24,041 (13.16%)
Length of Stay (days; mean, SD)	4.09 (3.53)
***Facility-Level Characteristics***	
Teaching Status	
No	148,656 (81.38%)
Yes	31,355 (17.17%)
To Be Determined	2655 (1.45%)
Academic Status	
No	161,362 (88.34%)
Yes	21,304 (11.66%)
Urban/Rural Status	
Rural	29,964 (16.40%)
Urban	152,702 (83.60%)
Ownership Status	
Government—Federal	567 (0.31%)
Government—Hospital District or Authority	11,565 (6.33%)
Government—Local	4342 (2.38%)
Government—State	971 (0.53%)
Physician	271 (0.15%)
Proprietary	11,771 (6.44%)
Voluntary Non-Profit (Church)	26,489 (14.50%)
Voluntary Non-Profit (Private)	116,090 (63.55%)
Voluntary Non-Profit (Other)	10,600 (5.80%)
Size (Bed Count)	
[1, 100]	18,437 (10.09%)
(100, 200]	30,232 (16.55%)
(200, 300]	37,147 (20.34%)
(300, 400]	32,176 (17.61%)
(400, 500]	19,970 (10.93%)
(500, 600]	14,741 (8.07%)
(600, 700]	9347 (5.12%)
(700, 800]	9046 (4.95%)
(800, 900]	5789 (3.17%)
(900, 1000]	2355 (1.29%)
(1000, 2000]	3426 (1.88%)
Case Mix Index (rounded)	
0	5931 (3.25%)
1	75,516 (41.34%)
2	100,970 (55.28%)
3	242 (0.13%)
4	7 (0.01%)
Census Region	
East—North Central	36,144 (19.79%)
East—South Central	15,916 (8.71%)
Middle Atlantic	23,642 (12.94%)
Mountain	9886 (5.41%)
New England	4166 (2.28%)
Pacific	13,568 (7.43%)
South Atlantic	49,091 (26.87%)
West—North Central	9327 (5.11%)
West—South Central	20,926 (11.46%)
Admission Month	
January	21,438 (11.74%)
February	21,040 (11.52%)
March	22,458 (12.29%)
April	16,411 (8.98%)
May	14,129 (7.73%)
June	12,014 (6.58%)
July	10,490 (5.74%)
August	9708 (5.31%)
September	10,815 (5.92%)
October	11,463 (6.28%)
November	13,056 (7.15%)
December	19,644 (10.75%)

^1^ AHRQ = Agency for Healthcare Research and Quality. ^2^ ICF = Intermediate Care Facility. ^3^ SNF = Skilled Nursing Facility. ^4^ MS-DRG = Medicare Severity Diagnosis-Related Group. ^5^ MCC = Major Complication/Comorbidity. ^6^ CC = Complication/Comorbidity.

**Table 2:** Poisson additive model incidence rate ratios (IRRs) and corresponding 95% confidence intervals (CIs), as well as *p*-values, for patient characteristics.

Patient-Level Characteristics	IRR	95% CI	*p*-Value
Intercept	16.27	16.19–16.35	<0.0001
Age (Reference: Over 84)			
<1	0.36	0.35–0.36	<0.0001
1–4	0.42	0.41–0.42	<0.0001
5–9	0.46	0.45–0.47	<0.0001
10–14	0.53	0.52–0.55	<0.0001
15–19	0.57	0.56–0.59	<0.0001
20–24	0.68	0.66–0.69	<0.0001
25–34	0.76	0.75–0.76	<0.0001
35–44	0.90	0.89–0.91	<0.0001
45–54	1.02	1.01–1.03	<0.0001
55–59	1.07	1.06–1.08	<0.0001
60–64	1.09	1.08–1.10	<0.0001
65–69	1.05	1.04–1.05	<0.0001
70–74	1.07	1.06–1.07	<0.0001
75–79	1.07	1.06–1.07	<0.0001
80–84	1.05	1.05–1.06	<0.0001
Sex (Reference: Female)			
Male	1.01	1.00–1.01	<0.0001
Unknown	1.03	0.96–1.10	0.3900
Race (Reference: White)			
American Indian	1.05	1.03–1.06	<0.0001
Asian	0.85	0.84–0.86	<0.0001
Black	0.99	0.99–1.00	<0.0001
Pacific Islander	0.89	0.87–0.90	<0.0001
Other	0.93	0.92–0.93	<0.0001
Unknown	0.87	0.87–0.88	<0.0001
AHRQ ^1^ Overall Tract Summary	0.95	0.94–0.95	<0.0001
Primary Payor (Reference: Medicare Traditional)			
Charity	0.71	0.70–0.73	<0.0001
Commercial Indemnity	0.86	0.85–0.86	<0.0001
Direct Employer Contract	0.86	0.83–0.89	<0.0001
Managed Care Capitated	0.86	0.83–0.88	<0.0001
Managed Care Non-Community-Acquired Pneumonia	0.81	0.80–0.81	<0.0001
Medicaid–Managed Care Community-Acquired Pneumonia	0.87	0.86–0.88	<0.0001
Medicaid–Managed Care Non-Community-Acquired Pneumonia	0.91	0.90–0.91	<0.0001
Medicaid Traditional	0.94	0.93-0.95	<0.0001
Medicare–Managed Care Community-Acquired pneumonia	0.99	0.99–1.00	0.1072
Medicare–Managed Care Non-Community-Acquired pneumonia	0.99	0.99–0.99	<0.0001
Other Government Payors	0.94	0.93–0.95	<0.0001
Self-Pay	0.73	0.73–0.74	<0.0001
Other	0.87	0.86–0.88	<0.0001
Point of Origin (Reference: Non-Healthcare Facility)			
Clinic	0.96	0.96–0.97	<0.0001
Referred from a Hospital (Different Facility)	1.00	0.99–1.01	0.7277
Referred from Department Unit in Same Hospital; Separate Claim	0.87	0.85–0.88	<0.0001
Referred from Health Facility	0.97	0.95–0.98	<0.0001
Referred from Skilled Nursing Facility or Intermediate Care Facility	1.02	1.01–1.03	<0.0001
Other	0.97	0.93–1.01	0.1328
Information Not Available	0.98	0.96–0.99	0.0081
Patient Discharge Status (Reference: Discharged to Home or Self-Care)			
Discharged to Home Health Organization	1.11	1.11–1.12	<0.0001
Discharged to Hospice–Home	1.22	1.21–1.23	<0.0001
Discharged to Hospice–Medical Facility	1.26	1.25–1.28	<0.0001
Discharged/Transferred to Intermediate Care Facility	1.07	1.06–1.09	<0.0001
Discharged/Transferred to Other Facility	1.13	1.12–1.15	<0.0001
Discharged/Transferred to Psychiatric Hospital	0.98	0.96–1.01	0.3130
Discharged/Transferred to Skilled Nursing Facility	1.12	1.12–1.13	<0.0001
Discharged/Transferred to Swing Bed	1.07	1.05–1.10	<0.0001
Discharged/Transferred to Other Health Institute not in List	1.04	1.00–1.07	0.0294
Discharged/Transferred to a Long-Term Care Hospital	1.15	1.13–1.17	<0.0001
Discharged/Transferred to Another Rehabilitation Facility	1.13	1.12–1.14	<0.0001
Expired	1.32	1.31–1.33	<0.0001
Left Against Medical Advice	1.07	1.06–1.08	<0.0001
Other	1.10	1.08–1.12	<0.0001
ICD-10-CM Principal Diagnosis (Reference: J18—Pneumonia, unspecified organism)			
J09: Influenza due to certain identified influenza viruses	0.87	0.85–0.89	<0.0001
J10: Influenza due to other identified influenza virus	0.93	0.93–0.93	<0.0001
J11: Influenza due to unidentified influenza virus	0.91	0.90–0.92	<0.0001
J12: Viral pneumonia, not elsewhere classified	1.00	1.00–1.01	0.6140
J13: Pneumonia due to streptococcus pneumoniae	0.95	0.94–0.97	<0.0001
J14: Pneumonia due to Hemophilus influenzae	1.01	0.99–1.03	0.3320
J15: Bacterial pneumonia, not elsewhere classified	1.00	0.99–1.00	0.2477
J16: Pneumonia due to other infectious organisms, not elsewhere classified	0.96	0.94–0.99	0.0020
R09: Other symptoms and signs involving the circulatory and respiratory system—as a primary diagnosis code	1.15	1.11–1.19	<0.0001
Other	1.08	1.00–1.17	0.0489
MS-DRG ^2^ Code (Reference: 193—Simple Pneumonia or Pleurisy with MCC ^3^)			
194: Simple Pneumonia and Pleurisy with CC ^4^	0.89	0.89–0.89	<0.0001
195: Simple Pneumonia and Pleurisy without CC/MCC	0.60	0.60–0.61	<0.0001
Log of Length of Stay (Spline coefficients) χ^2^ = 25,873 (*p* < 0.0001)			
Model Fit Deviance explained: 43.5%; Adj. R-squared 0.392			

^1^ AHRQ = Agency for Healthcare Research and Quality. ^2^ MS-DRG = Medicare Severity Diagnosis-Related Group. ^3^ MCC = Major Complication/Comorbidity. ^4^ CC = Major Complication/Comorbidity.

**Table 3:** Summary of linear regression analysis to estimate excess coding intensity adjusted for facility-level characteristics.

Facility-Level Characteristics	Estimate	SE ^1^	*p*-Value
Intercept	−0.38	0.10	0.0002
Teaching Status (Reference: No)			
Yes	0.56	0.06	<0.0001
TBD ^2^	0.02	0.18	0.8601
Academic Status (Reference: No)			
Yes	−0.47	0.07	<0.0001
Urban/Rural Status (Reference: Urban)			
Rural	0.04	0.04	0.4191
Ownership Status (Reference: Voluntary Non-Profit-Private)			
Federal	−0.06	0.27	0.8309
Hospital District or Authority	−0.15	0.06	0.0130
Local	−2.07	0.10	<0.0001
Government—State	−1.44	0.20	<0.0001
Physician	−0.32	0.37	0.3812
Proprietary	−0.74	0.06	<0.0001
Voluntary Non-Profit—Church	−0.15	0.04	0.0004
Voluntary Non-Profit—Other	−0.27	0.06	<0.0001
Size (Bed Count) (Reference: 1, 100)			
(100, 200)	0.02	0.06	0.7529
(200, 300)	0.04	0.06	0.4527
(300, 400)	0.29	0.06	<0.0001
(400, 500)	0.31	0.07	<0.0001
(500, 600)	0.42	0.07	<0.0001
(600, 700)	−0.21	0.09	0.0177
(700, 800)	0.51	0.09	<0.0001
(800, 900)	0.16	0.11	0.1433
(900, 1000)	0.26	0.15	0.0792
(1000, 2000)	0.57	0.12	<0.0001
Region (Reference: South Atlantic)			
North Central	−0.01	0.04	0.8572
East South Central	−0.68	0.06	<0.0001
Middle Atlantic	−1.59	0.05	<0.0001
Mountain	−0.55	0.07	<0.0001
New England	−1.45	0.10	<0.0001
Pacific	−1.11	0.06	<0.0001
West North Central	−0.43	0.07	<0.0001
West South Central	−0.36	0.05	<0.0001
Case Mix Index (Reference: 0)			
1	0.80	0.08	<0.0001
2	0.84	0.08	<0.0001
3	2.34	0.40	<0.0001
4	−1.45	2.26	0.5225

^1^ SE = Standard error. ^2^ TBD = To be determined.

## Data Availability

Data were provided by Premier, Inc., and is proprietary but accessible for purchase.

## Supplementary Materials

The following supporting information can be downloaded at: https://www.mdpi.com/article/10.3390/diagnostics12061495/s1, Figure S1: Histogram of the outcome additional diagnoses across patient visit; Figure S2: Histograms for the covariate length of stay; Figure S3: Proportion of primary payor by patient race; Figure S4: Fitted spline between log-transformed length of stay and expected number of additional diagnoses; Figure S5: Distribution of excess coding intensity; Figure S6: Effect plot of excess coding intensity for urban and rural facilities; Figure S7: Effect plot of excess coding intensity by facility ownership status; Figure S8: Effect plot of excess coding intensity by facility teaching status; Figure S9: Effect plot of excess coding intensity by facility academic status; Figure S10: Effect plot of excess coding intensity by facility size (i.e., bed capacity); Figure S11: Effect plot of excess coding intensity by the U.S. Census Bureau regional division; Figure S12: Effect plot of excess coding intensity by facility Case Mix Index (CMI); Figure S13: Heat map of average adjusted excess coding intensity across admission month and U.S. Census Bureau regional division; Figure S14: Dot plot of sorted average-adjusted excess coding intensity (unexplained excess coding intensity) by facility.
