# Supplementary material for: Diagnostic Coding Intensity among a Pneumonia Inpatient Cohort Using a Risk-Adjustment Model and Claims Data: A U.S. Population-Based Study
Source: Diagnostics (Basel). 2022 Jun 19;12(6):1495. doi: 10.3390/diagnostics12061495 (PMC9221672; doi:10.3390/diagnostics12061495)
Supplement: Supplementary file 1 [file diagnostics-12-01495-s001.zip › diagnostics-1768816-supplementary.pdf]

| Figures | Page(s) |
|---|---|
| S1. Histogram of the outcome additional diagnoses across patient-visits | 2 |
| S2. Histograms for the covariate length of stay | 3 |
| S3. Proportion of primary payor by patient race | 4 |
| S4. Fitted spline between log-transformed length of stay and expected number of additional diagnoses | 5 |
| S5. Distribution of excess coding intensity | 6 |
| S6. Effect plot of excess coding intensity for urban and rural facilities | 7 |
| S7. Effect plot of excess coding intensity by facility ownership status | 8 |
| S8. Effect plot of excess coding intensity by facility teaching status | 9 |
| S9. Effect plot of excess coding intensity by facility academic status | 10 |
| S10. Effect plot of excess coding intensity by facility size (i.e., bed capacity) | 11 |
| S11. Effect plot of excess coding intensity by U.S. Census Bureau regional division | 12 |
| S12. Effect plot of excess coding intensity by facility case mix index (CMI) | 13 |
| S13. Heat map of average adjusted excess coding intensity across admission month and U.S. Census Bureau regional division. | 14 |
| S14. Dot plot of sorted average adjusted excess coding intensity (unexplained excess coding intensity) by facility. | 15 |

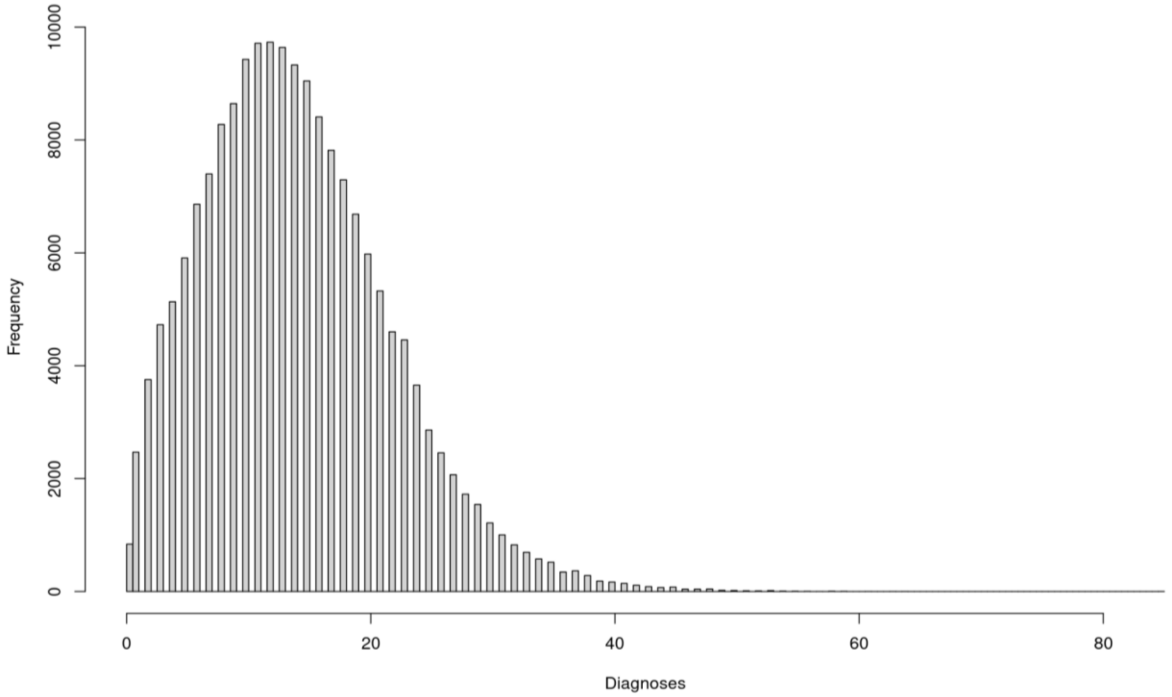


**Figure S1.** Histogram of additional diagnoses across patient-visits.

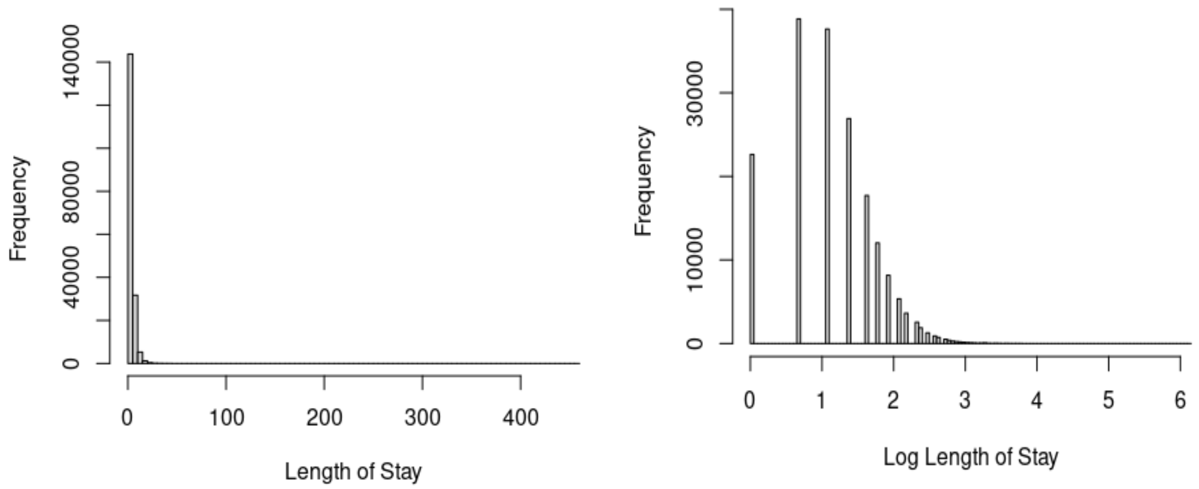


(**a**) (**b**)

**Figure S2.** Histograms for the covariate length of stay. Panel (a) shows the distribution in original units, and panel (b) shows the log-transformed histogram.

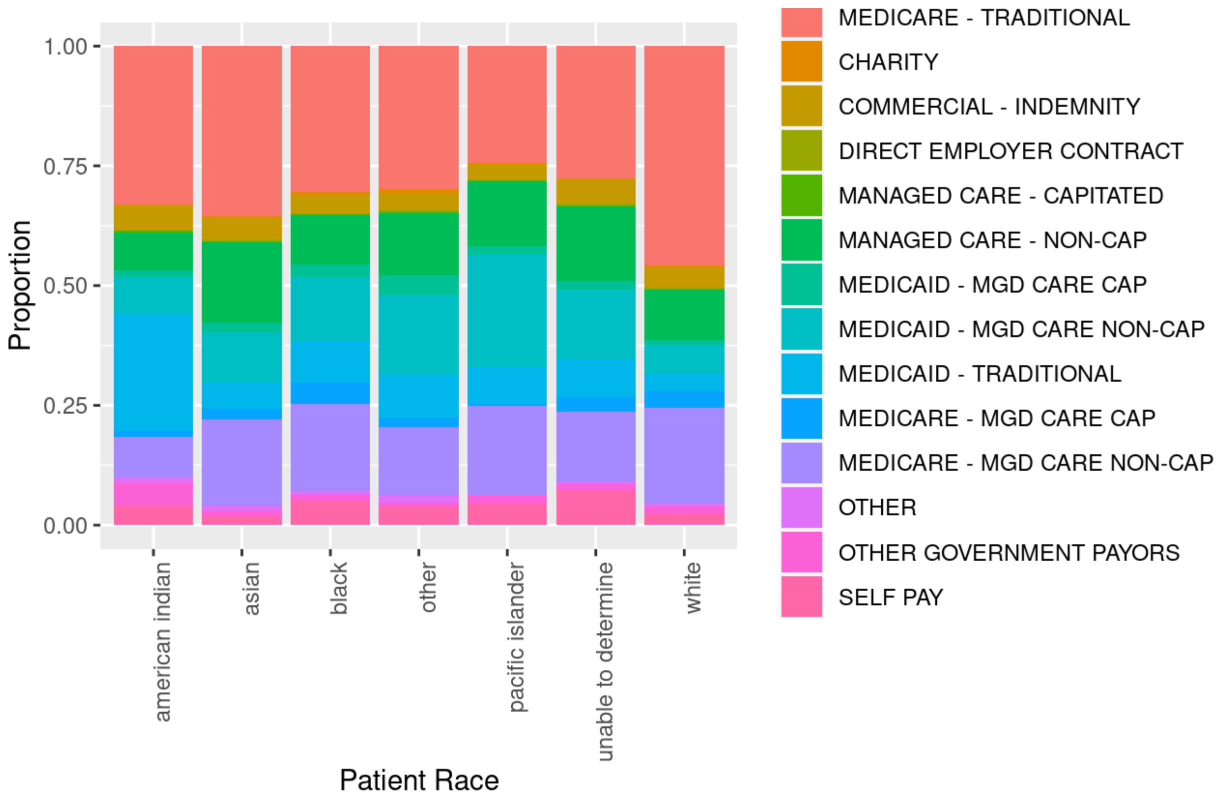


**Figure S3.** Proportion of primary payor by patient race.

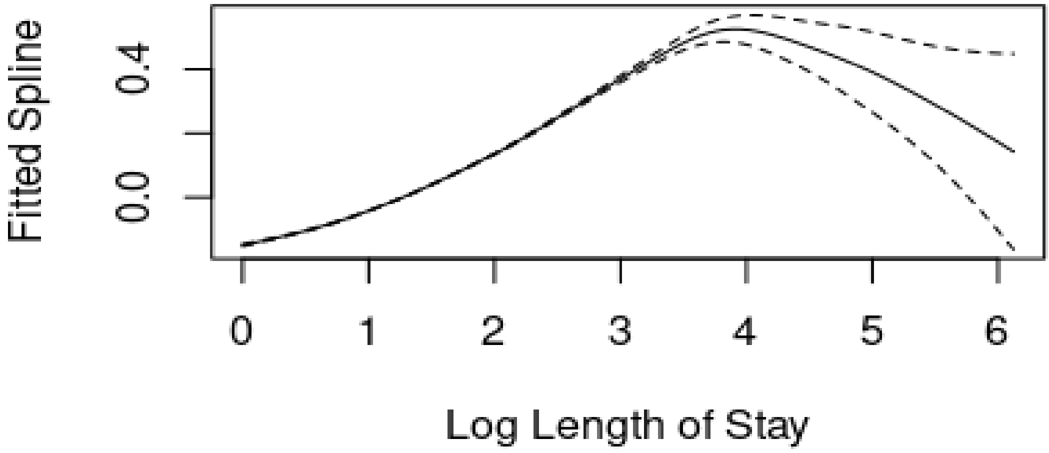


**Figure S4.** Fitted spline between the log-transformed length of stay and the expected number of additional diagnoses.

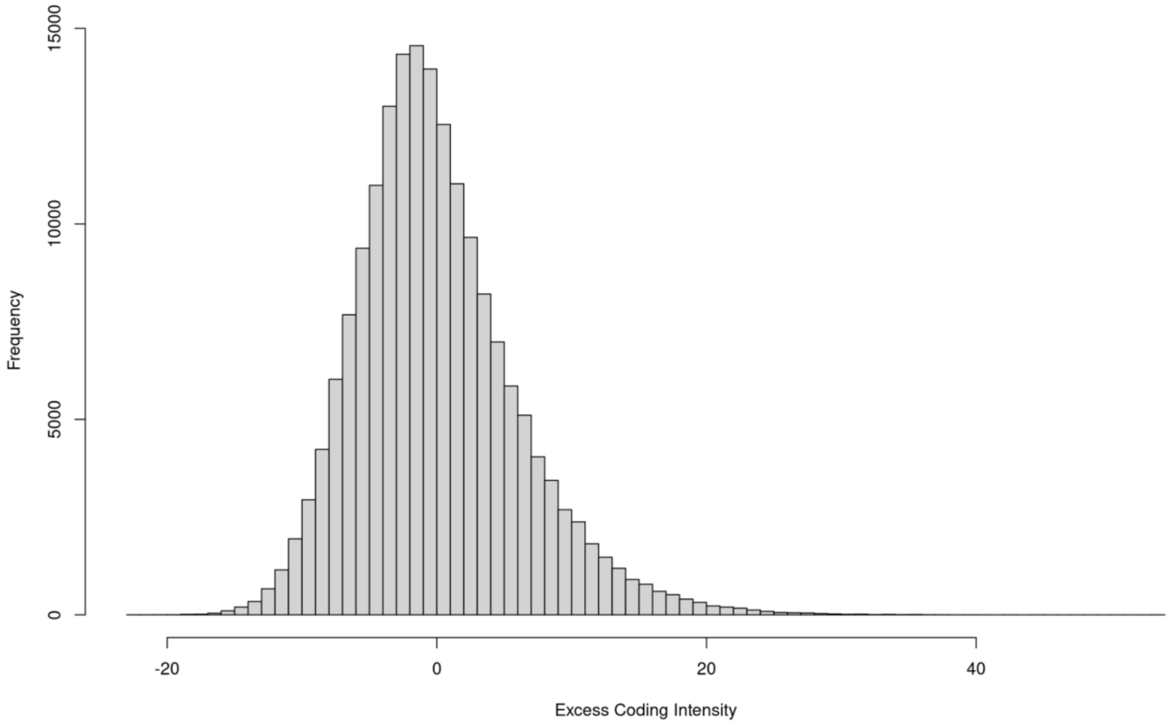


**Figure S5.** Distribution of excess coding intensity.

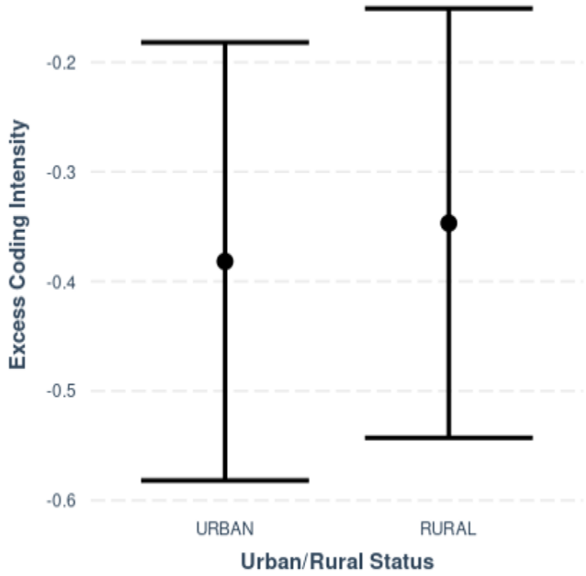


**Figure S6.** Effect plot of excess coding intensity for urban and rural facilities.

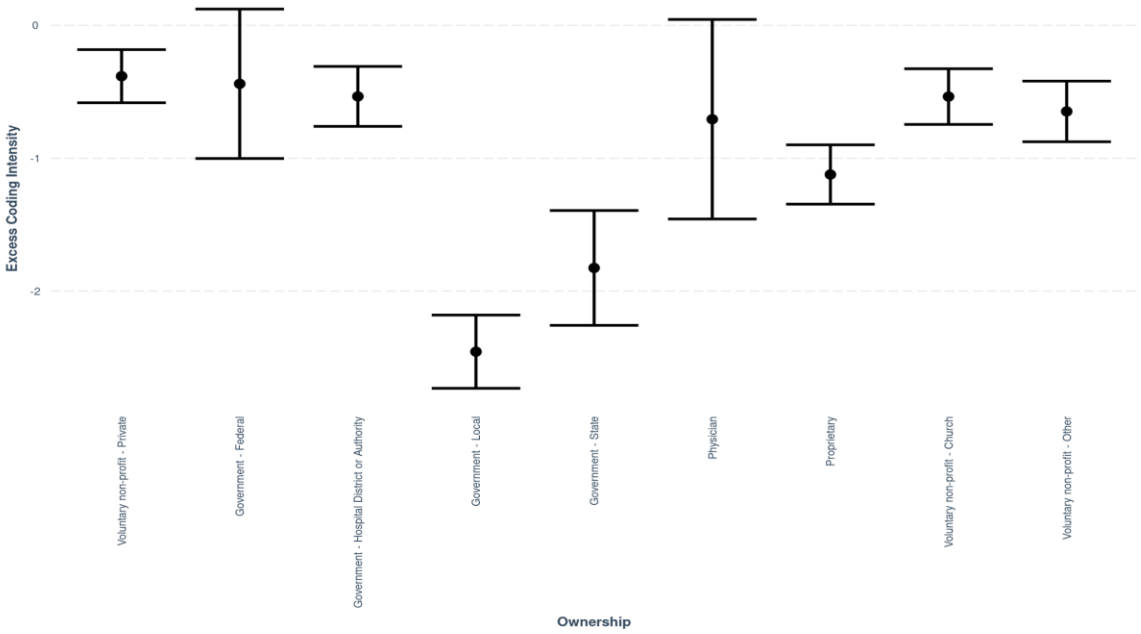


**Figure S7.** Effect plot of excess coding intensity by facility ownership status.

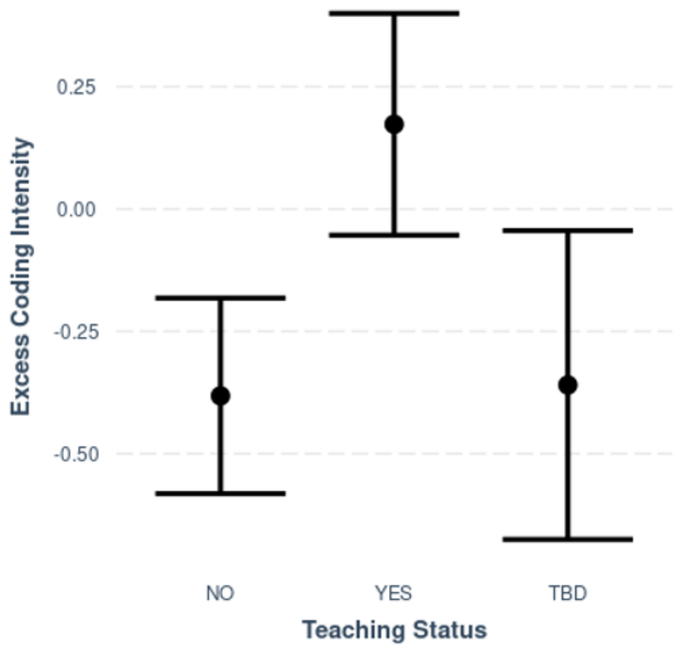


**Figure S8.** Effect plot of excess coding intensity by facility teaching status. TBD corresponds to facilities with a teaching status yet to be defined.

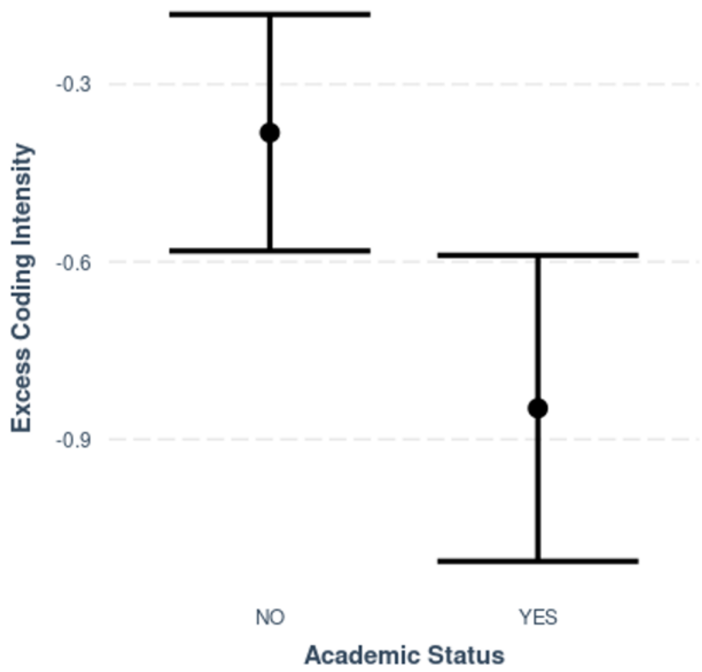


**Figure S9.** Effect plot of excess coding intensity by facility academic status.

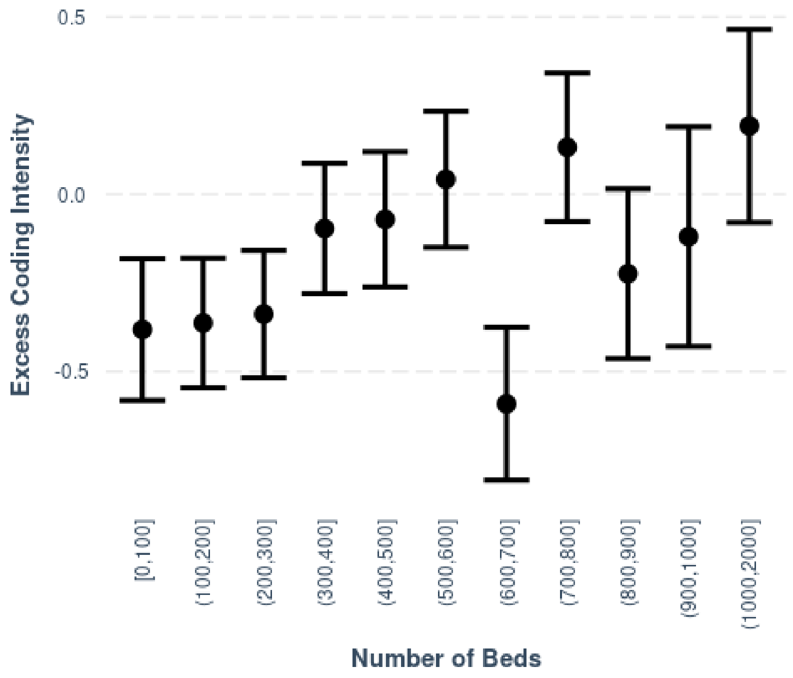


**Figure S10.** Effect plot of excess coding intensity by facility size (i.e., bed capacity).

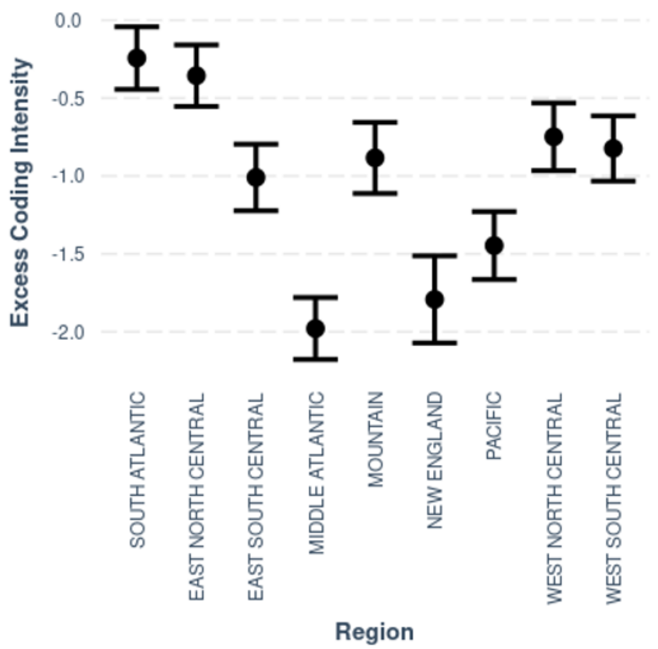


**Figure S11.** Effect plot of excess coding intensity by U.S. Census Bureau regional division.

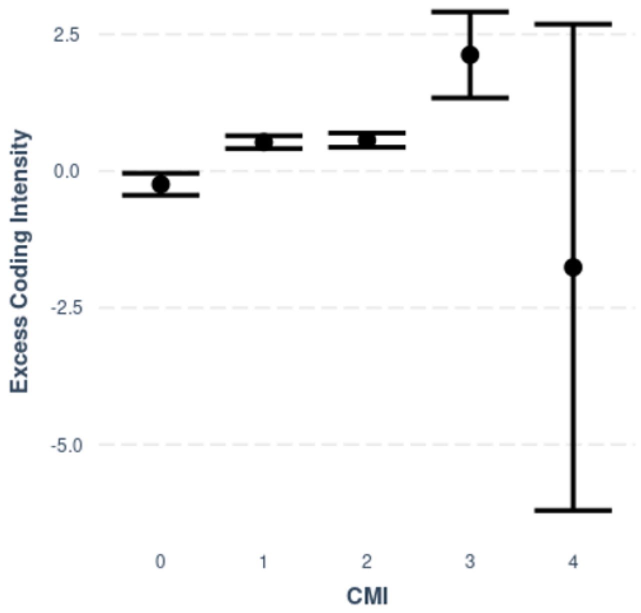


**Figure S12.** Effect plot of excess coding intensity by facility case mix index (CMI).

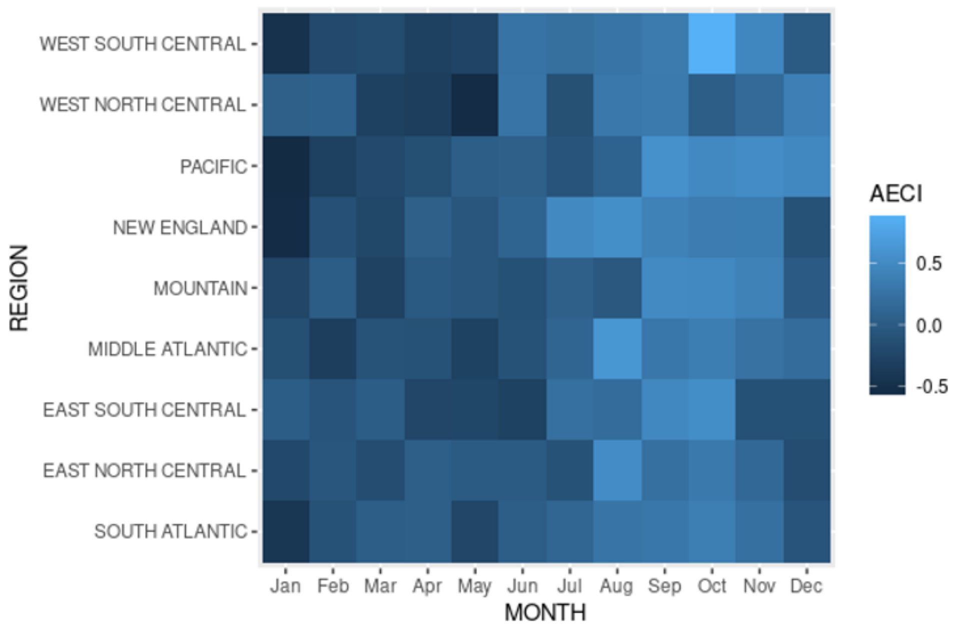


**Figure S13.** Heat map of average adjusted excess coding intensity across admission month and U.S. Census Bureau regional division.

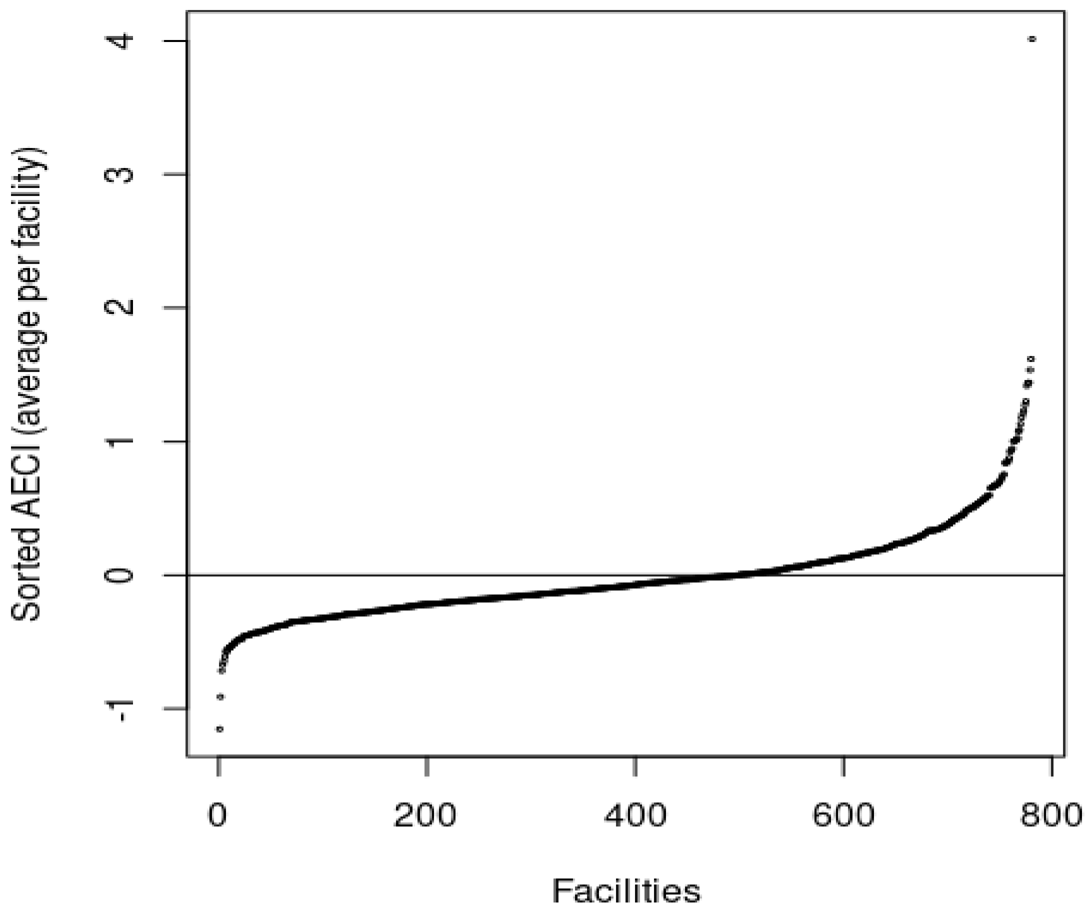


**Figure S14.** Dot plot of sorted average adjusted excess coding intensity (unexplained excess coding intensity) by facility.
